# The drug transporter *ABCB1* c.3435C>T SNP influences artemether–lumefantrine treatment outcome

**DOI:** 10.1186/s12936-017-2006-6

**Published:** 2017-09-21

**Authors:** Kinanga Kiaco, António Sebastião Rodrigues, Virgílio do Rosário, José Pedro Gil, Dinora Lopes

**Affiliations:** 1 Unidade de Parasitologia Médica, Instituto de Higiene e Medicina Tropical, Universidade Nova de Lisboa, Lisbon, Portugal; 2 Global Health and Tropical Medicine (GHTM), Instituto de Higiene e Medicina Tropical, Universidade Nova de Lisboa, Lisbon, Portugal; 3 Serviços de Saúde das Forças Armadas Angolanas, Estado Maior General das Forças Armadas, Luanda, Angola; 4 Centre for Toxicogenomics and Human Health, Genetics, Oncology and Human Toxicology, Universidade Nova de Lisboa, Lisbon, Portugal; 5 Drug Resistance Unit, Division of Pharmacogenetics, Department of Physiology and Pharmacology, Karolinska Institutet, Stockholm, Sweden; 6 Center for Biodiversity, Functional and Integrative Genomics, Faculdade de Ciências, Universidade de Lisboa, Lisbon, Portugal

**Keywords:** Human polymorphism, *CYP450*, *MDR1*, Artemether–lumefantrine, Angola

## Abstract

Malaria treatment performance is potentially influenced by pharmacogenetic factors. This study reports an association study between the *ABCB1 c.3435C*>*T*, CYP3A4*1B (g.-392A>G), CYP3A5*3 (*g.6986A*>*G*) SNPs and artemether + lumefantrine treatment outcome in 103 uncomplicated malaria patients from Angola. No significant associations with the CYP3A4*1B and CYP3A5*3 were observed, while a significant predominance of the *ABCB1 c.3435CC* genotype was found among the recurrent infection-free patients (p < 0.01), suggesting a role for this transporter in AL inter-individual performance.

## Background

Artemisinin combination therapy (ACT) has contributed to the remarkable decline by 48% in the malaria mortality rate between 2000 and 2015 [1]. The disease remains a major public health challenge, causing over 400,000 deaths annually, partly due to underperforming treatments. The success of malaria treatment depends on many factors, not least inter-individual pharmacokinetic differences, which are potentially influenced by patient pharmacogenetic background [2].

Artemether–lumefantrine (AL) is the most adopted antimalarial by national malaria control programs worldwide. In Angola, it represents the first-line treatment of choice for uncomplicated malaria.

Upon AL oral administration, artemether shows an elimination half-life of 1–3 h, CYP3A4 being the main enzyme involved in its conversion towards the (also active) dihydroartemisinin (DHA) metabolite [3]. Both artemether and DHA act rapidly to clear malaria parasites from circulation, reducing asexual parasite mass [4]. The lumefantrine partner has a half-life of 3–6 days and is responsible for the elimination of parasites remaining from the artemisinin ‘first impact’ action, while preventing recurrent malaria parasitaemia [5]. Only <10% of the absorbed LUM is biotransformed towards the active desbutyl-benflumetol (DBB) metabolite, mainly by CYP3A enzymes [6].

Both lumefantrine and DHA are essentially eliminated through the bile. In the apical biliocanalicular membrane of the hepatocyte, the ABCB1 (MDR1/Pgp) ATP-binding cassette (ABC) transporter is a major biliary efflux pump, particularly for lipophilic substrates, as lumefantrine [7, 8]. Significant inter-individual variation in drug exposure is known for both artemisinin and lumefantrine, suggesting the potential importance of *CYP3A4* and *ABCB1* pharmacogenetic characteristics influencing AL performance.

A previous attempt to correlate lumefantrine pharmacokinetic (PK) parameters with *CYP3A4* and *ABCB1* tag SNPs, particularly the promoter located *g.*-*392A*>*G* in the former (CYP3A4*1B) and the synonymous *c.3435C*>*T* in the latter, did not yield significant associations [9], having prompted the natural conclusion that such variation had a negligible effect [9]. Possible positive associations were anyway recently suggested for *ABCB1 c.3435C*>*T* with altered LUM exposure among HIV positive subjects under Efavirenz based therapy.

In the present work, we hypothesized that small pharmacokinetic differences might have observable pharmacodynamic consequences, the parasite reaction being a more sensitive parameter of the individual pharmacogenetic influence. Parasite clearance on day 3 post-treatment, recurrent infection prevalence and the 28-day cure rate endpoint of adequate clinical and parasitologic response (ACPR) were herein used as parameters to assess the effect of the patient pharmacogenetic status on AL in vivo anti-parasite performance. To test this hypothesis, a previously performed AL efficacy trial was analysed.

## Methods

### Patients

One-hundred and three unrelated patients with microscopy confirmed (1000–100,000 asexual parasites/µL) uncomplicated *Plasmodium falciparum* malaria involved in an AL (Coartem^®^, Novartis AG, Basel) efficacy trial in the Luanda region, Angola, conducted during the 2011–2013 period [10]. Briefly, upon informed written consent by the participant or their guardians, patients were treated with weight-adjusted, six-dose AL in 3 days, in accordance with national guidelines [11, 12]. Clinical assessment was performed at D_2_, D_3_, D_7_, D_14_, D_21_ and D_28_. At each time-point, thick blood films were examined for the presence of parasites and a capillary blood sample obtained for PCR analysis.

Ethical approval was obtained from the Angolan National Public Health Institute/Ministry of Health Ethics Committee. All procedures followed the latest version of the Declaration of Helsinki.

### Molecular genotyping

Capillary blood sample were collected on filter paper (FTA^®^ Classic Card, Whatman). DNA extraction was done by phenol–chloroform methods. The *ABCB1 c.3435C*>*T, CYP3A4* *g.*-*392C*>*G* and *CYP3A5* *g.6986A*>*G* SNPs were analysed by established PCR–RFLP protocols [13]. Presence of parasitaemia was further tested through the *pfmsp2* PCR amplification of all samples at all time spots [10]. Allele frequencies of the analysed SNPs were compared between groups of patients in accordance with two different treatment outcome phenotypes: (a) *pfmsp2* positive PCR by D_3_, informative concerning the artemisinin partner performance, in accordance with WHO guidelines; and, (b) *pfmsp2* positive PCR during the 28-day follow up (lumefantrine prophylactic effect). The number of clinical failure events (PCR-corrected recrudescence) was too small to warrant their specific analysis. All recrudescences were included in the general recurrence group.

Genotyping data for the parasite *pfmdr1*N86Y SNP, a well-established factor in parasite in vivo response to AL [14, 15], was available through previously performed PCR–RFLP methods [10].

### Statistical analysis

Data on SNPs were analysed using IBM SPSS version 23. Chi square (*χ* ^2^) test and Z statistics were used to determine significant differences between proportions (Graphpad Prism^®^ 7, Graphpad Software Inc, La Jolla, CA, USA). Five individual associations were herein tested: recurrence vs CYP3A4*1B allele status, recurrence *vs ABCB1 c3435C*>*T* SNP status, recurrence vs CYP3A5*3 SNP status, D_3_ positivity vs CYP3A4*1B SNP status, and D_3_ positivity vs *ABCB1 c.3435C*>*T* SNP status). Accordingly, a Bonferroni-corrected significance threshold at p < 0.01 was considered for these tests. Multivariate Log-linear analysis was performed in order to specifically investigate associations of key three categorical variables: (a) D28 follow-up outcome (recurrence), (b) *ABCB1/MDR1 c.3435C*>*T* genotype, and (c) *pfmdr1* N86Y status of the initial infection. The multiple testing was Benjamini–Hochberg corrected, assuming a 10% false discovery rate (q).

## Results

The 28-day PCR-corrected cure rate was 91.3%. On D_3_, 46.6% (n = 48) had positive PCR. During the 28-day follow up, 29/103 patients experienced recurrent parasitaemia, as detected through PCR. Ninety-eight patients were successfully analysed for the *CYP3A4* -*392A*>*G* SNP. The genotype frequency in this Angolan population was 0.112 (0.060–0.196, 11/98) for the wild type (*g.*-*392AA*), 0.541 (0.438–0.641, 53/98) minor allele homozygous (*g.*-*392GG*) and 0.347 (0.438–0.641, 34/98) for the heterozygous (*g.*-*392AG)*. The population was found in Hardy–Weinberg equilibrium for this locus (p > 0.05).

The patient CYP3A4*1B genotype was not found to be significantly associated with either D3 parasite positivity (*χ* ^2^ = 5.493, df = 1, p = 0.019) (Table 1) or treatment outcome upon the 28-day follow up (*χ* ^2^ = 2.378, df = 1, p = 0.123) (Table 2).

**Table 1:** *CYP3A4*-392A>G and ABCB1 3435C>T genotype frequencies and D_3_ PCR positivity (IC95%)

Gene/SNP	Genotype frequencies (IC 95%)					
	D3 positive			D3 negative		
CYP3A4*1B	AA	AG	GG	AA	AG	GG
	3/48 (*0.062*; 0.016–0.182)	14/48 (*0.292*; 0.174–0.443)	31/48 (*0.646*; 0.440–0.775)	8/49 (*0.163*; 0.078–0.302)	21/49 (*0.429*; 0.291–0.577)	20/49 (*0.408*; 0.270–0.558)
*MDR1, c3435C>T*	CC	CT	TT	CC	CT	TT
	34/47 (*0.723*; 0.571–0.839)	9/47 (*0.192*; 0.097–0.337)	4/47 (*0.085*; 0.028–0.213)	41/51 (*0.804*; 0.665–0.897)	7/51 (*0.137*; 0.062–0.269)	3/51 (*0.059*; 0.015–0.172

NO significant associations were

*D3 positive* positive PCR by day 3; *D3 negative* negative PCR by day 3

**Table 2:** *CYP3A4*-392A>G and ABCB1 3435C>T genotype frequencies and risk of recurrency during the 28-day follow-up (IC95%)

Gene/SNP	Genotype frequencies (IC 95%)					
	Recurrence-free group			Recurrence group		
CYP3A4*1B	AA	AG	GG	AA	AG	GG
	8/71 (*0.113*; 0.053–0.215)	37/71 (*0.521*; 0.400–0.640)	26/71 (*0.366*; 0.258–0.490)	3/28 (*0.107*; 0.028–0.294)	10/28 (*0.357*; 0.193–0.559)	15/28 (*0.536*; 0.342–0.720)
*ABCB1* 3435C>T	CC	CT	TT	CC	CT	TT
	60/72* (*0.833*; 0.723–0.907)	10/72* (*0.077*; 0.072–0.245)	2/72* (*0.028*; 0.005–0.106)	17/29* (*0.586*; 0.391–0.760)	6/29* (*0.207*; 0.087–0.403)	6/29* (*0.207*; 0.087–0.403)
CYP3A5*3	AA	AG	GG	AA	AG	GG
	46/59 (*0.780*; 0.653–0.877)	11/59 (*0.186*; 0.097–0.309)	2/59 (*0.034*; 0.004–0.117)	17/25 (*0.680*; 0.465–0.851)	6/25 (*0.240*; 0.094–0.451)	2/25 (*0.080*; 0.010–0.260)

* Significant (p < 0.01)

*Recurrence-free group* without positive PCR during the follow-up days

*Recurrence group* with positive PCR during the follow-up days

Concerning the *ABCB1* c.3435C>T SNP, 101 patients were successfully tested. Genotype frequencies were 0.762 (0.667–0.841, 77/101) for the wild type (*c.3435CC*), 0.079 (0.035–0.150, 8/101) for the minor allele homozygous (*c.3435TT*) and 0.158, (0.093–0.244, 16/101) for the heterozygous (*c.3435CT*). The population was found in Hardy–Weinberg equilibrium for this locus (p > 0.05).

**Fig. 1:**
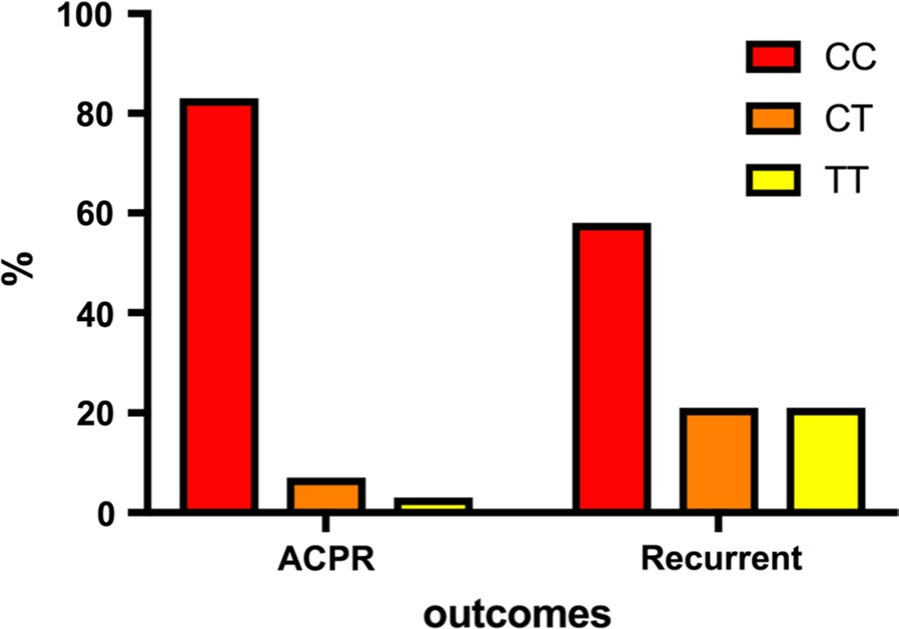
*ABCB1/MDR1 C3435T* ACPR vs recurrence experiencing group. *CC* wild type genotype, *CT* heterozygous genotype, *TT* mutant genotype

The *c.3435C*>*T* SNP was also not significantly associated with the D_3_ parasite PCR positivity (*χ* ^2^ = 0.883, df = 1, p = 0.347) (Table 1). On the other hand, *c.3435TT* genotypes were found to be significantly more frequent among patients experiencing recurrent events during follow-up (*χ* ^2^ = 6.9693, df = 1, p = 0.008) (Table 3, Fig. 1). These changes were further reflected on a significant increase in recurrence risk (OR = 10.59, 1.96–57.30, z-score = 2.739, p = 0.006) in this subgroup.

**Table 3:** Analysis of the association between ABCB1 c.3435C>T patient status and treatment outcome (*χ* ^2^ = 6.9693, df = 1, p = 0.008)

*ABCB1* c3435C>T	Recurrence status		Total
	Positive	ACPR	
CC	17	60	77
CT + TT *Total*	12	12	24
	29	72	*101*

During the completion of the present work, a new report has suggested CYP3A5 as a contributor to lumefantrine metabolism [16]. Following this lead, we have successfully analyzed CYP3A5*3 (*c.6986A*>*G*) in 84 samples, as this is the allele is the most investigated as having a robust deleterious effect in the expression of the gene [17]. Genotype frequencies were 0.750 (0.644–0.838, 63/84) for the wild type (*c.6986AA*), 0.048, (0.013–0.117, 4/84) for the heterozygous (*c.6986AG*) and 0.202 (0.123–0.304, 17/84) for the minor allele homozygous (*c.6986GG*). The sample population was found in Hardy–Weinberg equilibrium for this locus (p > 0.05).

As with CYP3A4*1B, no significant association was observed between the patient status for carrying a CYP3A5*3 alleles and the parasitological outcome during the 28 day follow up (*χ* ^2^ = 0.932, df = 1, p = 0.335)(Table 2).

Due to the size limitations of the study, multivariate analysis was limited to variables expected to interact concerning the clinical outcome under focus (D28 follow up positivity), namely the *ABCB1 c3435C*>*T* status and the *pfmdr1* N86Y status. A subset of 92 cases was available with complete data for these three variables (Table 4).

**Table 4:** Contingency table associated with the Log-linear multivariate analysis

*ABCB1* c3435C>T	*Pfmdr1*N86Y	Recurrence status		Total
		Positive (A1)	ACPR (A2)	
CC (B1)	86 N pure (C1)	8	41	49
	86 N/Y + 86Y (C2)	9	11	20
CT + TT (B2)	86 N pure (C1)	9	8	17
	86 N/Y + 86Y (C2)	2	4	6
*Total*		11	12	*92*

Upon the assumption of a false discovery rate of (q) of 10% for Benjamini–Hochberg multiple test correction, only two associations stood out as near the threshold of significance: the overall interaction between the three analysed variables (G^2^ = 10.84, df = 4, p = 0.0284 vs p (corrected) = 0.0286), and the association between recurrence during follow-up and the *ABCB1 c3435C*>*T* status (G^2^ = 8.36, df = 2, p = 0.0146 vs p (corrected) = 0.0143), after filtering the effect of the presence/absence of the *pfmdr1* N86 allele (Table 4). This analysis further suggests the importance of the *ABCB1 c3435C*>*T* SNP, albeit the limited number of samples available recommends caution in the interpretation of the results.

## Discussion

The present work focused on finding links between patient *CYP3A* and *ABCB1* pharmacogenetics and AL pharmacodynamics in vivo endpoints. D_3_ positivity was not significantly associated with the patient CYP3A4*1B status (Table 1), an observation that can be simply explained by the fact that DHA-the main artemether CYP3A4 metabolite-is also highly active against *P. falciparum* parasites. Pharmacogenetic driven variable rates of artemether to DHA bioconversion are likely not to be readily visible in terms of the artemisinin effect on the infection. As for the *ABCB1 c.3435C*>*T*, the negative observations possibly result from the specific capacity of this transporter to efflux more lypophilic compounds then the final phase II glucuronidated DHA extracted from the liver.

The CYP3A4*1B and CYP3A4*3 status were not seen to influence the risk of malaria recurrence. The AL post-treatment protective effect is essentially related with the action of lumefantrine, the long half-life partner. In regular conditions, it is expected that the large majority of lumefantrine is eliminated unchanged [18], a result supported by the previously observed modest effect of ketoconazole in interaction studies [6]. This means that any role of CYP3A4 and/or CYP3A5 will be limited to variations in this remain biotransformed fraction, which expected small size might have precluded its detection during the present works. One cannot nevertheless rule out the possibility that small changes in the concentrations of the resulting DBB metabolite might influence the treatment, in particular because of its higher anti-parasitic potency, as previously suggested [19]. Also, it is likely that scenarios of long-term CYP3A induction might increase the fraction of LUM metabolism-as potentially observed among patients under Efavirenz therapy [20]-and as such the role of this cytochrome P450s on lumefantrine elimination. Nevertheless, inside its size limitations and in this specific population, our study suggests a likely minor contribution of the CYP3A4*1B and CYP3A5*3 SNPs in modulating AL post-treatment prophylactic action.

A significant increase in the frequency of the *ABCB1 c.3435TT* genotype was found among patients suffering recurrent infections during the 28-day follow up, suggesting a role of the encoded P-glycoprotein. The synonymous *c.3435C*>*T* polymorphism has been proposed to be linked with altered rates of protein synthesis, leading to proteins that albeit having the same primary sequence, emerge from the process of translation with different tertiary conformations [21]. The functional effect of such changes in the P-glycoprotein seems to depend on the drug under consideration. In the present studies, a substantial predominance was found of the *ABCB1 c.3435CC* genotype among the recurrence-free patients, signalling an increased lumefantrine exposure associated with this genotype, which better shielded the recovering patients from new infections.

A shortcoming of the present study is the unavailability of pharmacokinetic data, namely D7 LUM levels, in order to have a complete pharmacokinetic/pharmacogenetic picture. Nevertheless, it is interesting to note that the present results are in agreement with recent data from Maganda et al. [20], where the *ABCB1 c.3435TT* genotype, was suggested to be associated with a significantly decreased D7 lumefantrine levels among patients undergoing malaria treatment with AL. Such an effect in drug exposure can explain the increase risk of these patients towards recurrent infection.

These data suggest lumefantrine as part of the group of ABCB1 substrates where this genotype is associated with increased drug exposure, probably due to a less efficient efflux. Other examples include tacrolimus [22, 23], silibinin [24], amlodipine [25], or in some studies, digoxine [26].

## Conclusion

By exploring potential pharmacodynamics/pharmacogenetic associations in anti-malarial therapy, this report shows a non-negligible influence of the host *ABCB1 c.3435C*>*T* SNP in the performance of artemether–lumefantrine. The present observations join other recent reports pointing for the importance drug transporter pharmacogenetics in ACT pharmacokinetics and pharmacodynamics [20, 27].
